# Observation of bound states in the continuum embedded in symmetry bandgaps

**DOI:** 10.1126/sciadv.abk1117

**Published:** 2021-12-22

**Authors:** Alexander Cerjan, Christina Jörg, Sachin Vaidya, Shyam Augustine, Wladimir A. Benalcazar, Chia Wei Hsu, Georg von Freymann, Mikael C. Rechtsman

**Affiliations:** 1 Department of Physics, The Pennsylvania State University, University Park, PA 16802, USA.; 2 Center for Integrated Nanotechnologies, Sandia National Laboratories, Albuquerque, NM 87185, USA.; 3 Physics Department and Research Center OPTIMAS, University of Kaiserslautern, D-67663 Kaiserslautern, Germany.; 4 Ming Hsieh Department of Electrical Engineering, University of Southern California, Los Angeles, CA 90089, USA.; 5 Fraunhofer Institute for Industrial Mathematics ITWM, 67663, Kaiserslautern, Germany.

## Abstract

In the past decade, symmetry-protected bound states in the continuum (BICs) have proven to be an important design principle for creating and enhancing devices reliant upon states with high-quality (*Q*) factors, such as sensors, lasers, and those for harmonic generation. However, as we show, current implementations of symmetry-protected BICs in photonic crystal slabs can only be found at the center of the Brillouin zone and below the Bragg diffraction limit, which fundamentally restricts their use to single-frequency applications. By microprinting a three-dimensional (3D) photonic crystal structure using two-photon polymerization, we demonstrate that this limitation can be overcome by altering the radiative environment surrounding the slab to be a 3D photonic crystal. This allows for the protection of a line of BICs by embedding it in a symmetry bandgap of the crystal. This concept substantially expands the design freedom available for developing next-generation devices with high-*Q* states.

## Teaser

Designing the photonic environment yields a mechanism for creating bound states in the continuum over a wide range of angles.

## INTRODUCTION

Over the past decade, bound states in the continuum (BICs) have emerged as an important design principle for creating systems with high-quality (*Q*) factor states to enhance light-matter interactions. BICs are states with theoretically infinite lifetimes despite the availability of a radiative continuum at the same frequency (*1*). By operating a system in the vicinity of a BIC in some generalized parameter space, arbitrarily large *Q* factors can be realized that allow the *Q* of the system to be tailored to the specific needs of the device. Although several different mechanisms can be used to create BICs in optical systems (*2*–*24*), the preponderance of interest has focused on using the structure’s symmetry to protect a BIC from radiating for two reasons: First, symmetry protection is predictive, and planar systems having 180° rotational symmetry about the *z* axis (*C*_2_) will generically have BICs. Second, no fine-tuning of the structure is necessary to adjust the BIC’s location in wave vector space, because the BIC is guaranteed to exist at normal incidence. Hence, symmetry-protected BICs in photonic crystal slabs and all-dielectric metasurfaces have been demonstrated to enable or enhance a wide variety of different applications, such as sensors (*25*–*27*), high-power on-chip lasers (*28*, *29*), vortex lasers (*30*, *31*), harmonic generation (*32*–*37*), and increased control over transmission and reflection spectra (*38*–*42*).

Despite these successes of using symmetry protection to create BICs for next-generation devices, current designs for achieving symmetry-protected BICs in slab geometries are fundamentally limited. As we rigorously prove in this work, if one is restricted to engineering structures within the slab, then symmetry-protected BICs always appear as isolated states that can only exist at normal incidence, below the Bragg diffraction limit. Thus, any application requiring a range of high-*Q* states, such as operating a sensor at multiple frequencies simultaneously or steering a laser beam using lasing modes in the same device with different in-plane wave vectors (*43*), cannot be realized with existing system designs using symmetry-protected BICs.

We show that these limitations of two-dimensional (2D) BICs can be overcome by designing the environment surrounding the slab, as opposed to the slab itself. We theoretically propose and experimentally realize a line of BICs in a “symmetry bandgap” by embedding a homogeneous slab in a 3D rectangular woodpile photonic crystal environment. Here, we define a symmetry bandgap as a wave vector–dependent frequency range along a high-symmetry line (or at a high-symmetry point) in which only a subset of the possible symmetry representations are present among states in the environment. Thus, a slab state with the appropriate symmetry representation inside a symmetry bandgap of the surrounding radiative environment is necessarily a symmetry-protected BIC. Our monolithic structure is fabricated entirely from photoresist polymer using two-photon polymerization (*44*, *45*) and characterized using angle-resolved Fourier transform infrared (FTIR) spectroscopy (*46*). The line of symmetry-protected BICs is directly observed as the vanishing linewidth of a resonance of the slab along a high-symmetry line of the system.

## RESULTS

### Symmetry restrictions for finding BICs

**Fig. 1.**
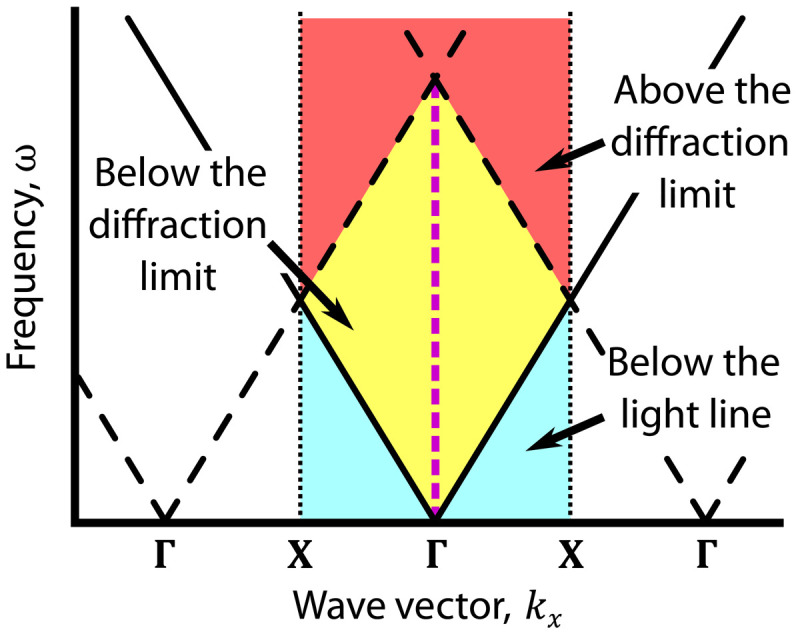
Radiative channel limits in the Brillouin zone for homogeneous, isotropic environments. Schematic showing the regions of the Brillouin zone in the *k_y_* = 0 plane that are below the light line (cyan), below the first Bragg diffraction limit (yellow), and above the first Bragg diffraction limit (red). High-symmetry points are marked, **Γ** = (0,0), and **X** = (π/*a*,0). For environments that are homogeneous and isotropic, symmetry-protected BICs can only exist at **Γ** and below the Bragg diffraction limit, indicated as the magenta dashed line. Only the first Brillouin zone is shaded as a reminder that slab resonances are only uniquely defined in **k**_∥_ up to a reciprocal lattice vector.

The capacity for a device to support symmetry-protected BICs can be viewed from the perspective of the environment: For a symmetry-protected BIC to exist, at least one symmetry representation of the device must be absent in the available radiative channels of the surrounding environment. As an example, consider the radiation from a resonance of a photonic crystal slab with in-plane wave vector **k**_∥_ = **Γ** = (0,0) into the surrounding air. At low frequencies, ω, conservation of in-plane momentum dictates that the only available radiative channels above the slab that the resonance can couple to are s- and p-polarized outgoing plane waves with **k** = (0,0, ω/*c*) (yellow region in Fig. 1). Both of these channels are odd with respect to rotation about the *z* axis by 180° (*C*_2_), as this rotation leaves **k** invariant but reverses the direction of the polarization. The same is true for the two available radiative channels below the slab. Thus, any photonic crystal slab that is *C*_2_ symmetric and surrounded by air will generically have states at **Γ** that are even with respect to *C*_2_ and cannot radiate because of this symmetry mismatch, i.e., these states are necessarily symmetry-protected BICs. In contrast, away from **Γ** but still in the interior of the first Brillouin zone, degenerate s- and p-polarized plane waves span all possible in-plane symmetry representations, so all the resonances of the slab will generally radiate at these **k**_∥_, which prohibits the formation of symmetry-protected BICs (see the Supplementary Materials).

In an isotropic and homogeneous environment, additional radiative channels become available above the Bragg diffraction limit (*47*), *n*ω/*c* >∣**k**_∥_ ± **b***_i_*∣ (red region in Fig. 1). Here, *i* = 1,2, **b***_i_* are the reciprocal lattice vectors of the slab, and *n* is the environment’s refractive index. These extra channels correspond to light acquiring additional momentum from the periodicity of the lattice as it radiates. Note that, along the first Brillouin zone’s boundary, frequencies that are above the light line, *n*ω/*c* >∣**k**_∥_∣, are necessarily above the Bragg diffraction limit. For such an environment, one can prove that these additional channels span all of the in-plane symmetries of the system for every **k**_∥_, prohibiting the formation of symmetry-protected BICs above the Bragg diffraction limit.

To give an abbreviated argument for this statement, consider a slab resonance with frequency ω and in-plane wave vector **k**_∥_ ≠ **Γ**, and an available radiative channel with wave vector **k** = (**k**_∥_, *k_z_* = (*n*^2^ω^2^/*c*^2^ − ∣**k**_∥_∣^2^)^(1/2)^). Assume that **k**_∥_ is a high-symmetry point where it may be possible for the slab resonance to have a symmetry representation that does not exist in the environment, and let *S* be an in-plane symmetry operation that leaves *S***k**_∥_ equivalent to **k**_∥_, i.e., *S***k**_∥_ = **k**_∥_ + ∑_*i* = 1,2_*m_i_***b***_i_* for *m_i_* ∈ ℤ. Then, there are two possibilities:

1) If *S***k**_∥_ = **k**_∥_, then for all 17 of the 2D space groups, *S* is a reflection or glide operation, which the resonance can be even or odd with respect to, but both of these possibilities are spanned by degenerate s- and p-polarized plane waves, as the two polarizations behave oppositely under reflections and glides.

2) If *S***k**_∥_ ≠ **k**_∥_, then these wave vectors correspond to orthogonal radiating plane waves, linear combinations of which necessarily span the possible in-plane symmetry representations of *S*.

Together, these two statements show that there will always be an available radiative channel for any slab resonance to couple to above the Bragg diffraction limit in a homogeneous and isotropic environment, precluding any symmetry-protected BICs from appearing. A rigorous proof using representation theory can be found in the Supplementary Materials. In summary, symmetry-protected BICs in periodic planar structures surrounded by homogeneous, isotropic environments can only be found at **k**_∥_ = **Γ** and below the Bragg diffraction limit (*n*ω/*c* < ∣**b**_1,2_∣ for this wave vector); no amount of engineering of the slab can overcome this limitation.

### Realizing BICs through environmental design

To realize symmetry-protected BICs anywhere else in the Brillouin zone of a planar structure, one is forced to break either isotropy or homogeneity of the radiative environment. Breaking isotropy (for example, by using a birefringent environment) lifts the degeneracy between s- and p-polarized plane waves, allowing for the possibility of a symmetry bandgap depending on the orientation of the underlying material’s crystalline axes (*10*, *12*).

Here, we break the homogeneity of the environment surrounding the slab by using a 3D photonic crystal as the radiative environment. In addition to breaking the degeneracy between s- and p-polarized plane waves (*15*), this also allows for radiative channels with *S***k**_∥_ ≠ **k**_∥_ to still be equivalent and correspond to the same radiative channel, as the environment now has discrete, not continuous, translational symmetry. Hence, a 3D photonic crystal environment can have symmetry bandgaps for **k**_∥_ ≠ **Γ**.

It may seem counterintuitive that, by reducing the symmetry of the environment from continuous to discrete translational symmetry, one somehow gains access to additional forms of symmetry protection in the slab, but patterning the environment provides two fundamental benefits beyond breaking the degeneracy between the two polarizations. First, using a 3D photonic crystal environment with discrete translational symmetry in *z* means that radiative channels have maximum frequency cutoffs, which is not the case in free space or homogeneous birefringent environments, and allows for symmetry bandgaps to appear even in high-frequency regions of the environment’s band structure. Second, in-plane discrete translational symmetries allow for the appearance of additional high-symmetry lines in the environment’s band structure along the boundary of the first Brillouin zone; there is nothing remarkable about the **Y−M** line in free space, but there is in the rectangular woodpile environment.

**Fig. 2.**
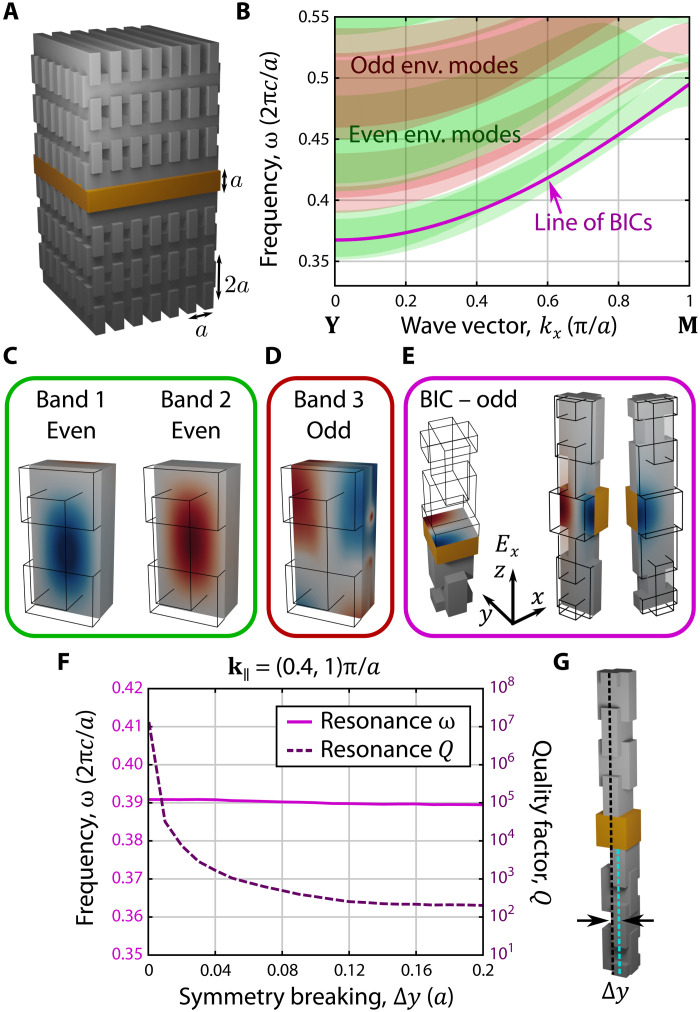
Symmetry-protected BIC in a slab embedded in a rectangular woodpile photonic crystal. (**A**) Schematic of a solid slab (orange) embedded in a rectangular woodpile photonic crystal environment (gray). (**B**) Projected-in-*k_z_* band structure of the woodpile environment (env.) along the **Y**−**M** high-symmetry line. Green (red) regions indicate where a projected band of the woodpile is even (odd) with respect to reflection about the *xz* plane. A line of BICs of the slab that are odd about this reflection symmetry is shown in purple. (**C** to **E**) *E_x_* modal profiles of the first three bands of the environment and one of the BICs at **k**_**∥**_*a*/π = (0.4,1), which are even (C) or odd (D and E) with respect to this reflection symmetry. (**F**) *Q* factor and resonance frequency of the BIC at **k**_**∥**_*a*/π = (0.4,1) as the upper and lower woodpile environments are displaced by Δ*y*, sketched in (**G**).

In particular, we embed a homogeneous slab inside a 3D rectangular woodpile photonic crystal environment (Fig. 2A). This changes the radiative channels of the environment to be the projected-in-*k_z_* bands of the photonic crystal environment, as the presence of the slab breaks translational symmetry in *z*. As the mostly evanescent tails of the slab’s resonances overlap with the periodic environment, no patterning of the slab is necessary for its resonances to form a photonic crystal slab band structure. Moreover, as the slab is homogeneous or, more generally, so long as the slab and the environment have commensurate in-plane lattice vectors, **k**_∥_ is conserved during radiation. For this photonic crystal environment, we find that, along the **Y**−**M** (**X**−**M**) high-symmetry line, where the system is reflection symmetric about the *xz* plane (*yz* plane), the two lowest-frequency radiative channels are both even with respect to this symmetry (Fig. 2, B to D. Hence, the rectangular woodpile exhibits a symmetry bandgap in its two lowest-frequency bands along these high-symmetry lines.

Within this symmetry bandgap, the states of the photonic slab with the opposite symmetry are necessarily symmetry-protected BICs, such as the state shown in Fig. 2 (B and E). Moreover, we can confirm that the exponential confinement of the state is due to its mismatched symmetry by displacing the rectangular woodpile environments above and below the slab to break the *xz* plane reflection symmetry of the whole structure. This displacement yields environments above and below the slab that no longer share the same reflection plane, so that their radiative channels necessarily span all the possible symmetries along the **Y**−**M** high-symmetry line, i.e., there is no longer a symmetry bandgap. Hence, this perturbation substantially decreases the *Q* of the slab resonance, as shown in Fig. 2 (F and G).

### Experimental observation

**Fig. 3.**
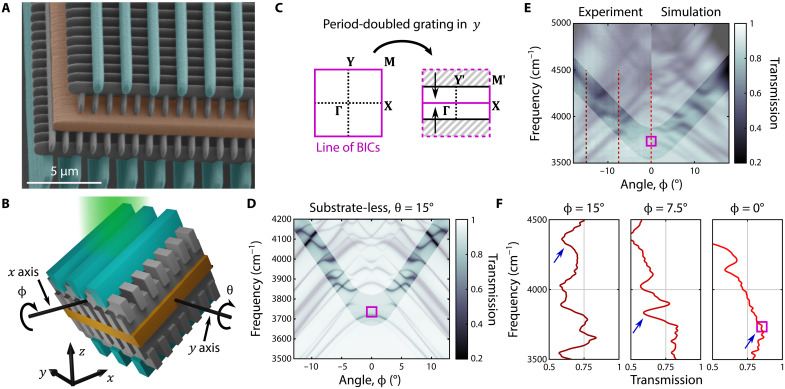
Observation of a symmetry-protected BIC away from Γ. (**A**) False color scanning electron microscope image of a photonic crystal slab (orange) embedded in a rectangular woodpile photonic crystal environment (gray) with period-doubled grating (cyan). (**B**) Sketch of the structure with angles θ and ϕ relevant for the measurements. (**C**) Schematic showing the folding of the first Brillouin zone due to the period-doubled grating. (**D**) Simulated transmission spectra of the substrate-less structure along ϕ. (**E**) Transmission spectra of the BIC structure (left, experiment; right, simulation) along ϕ. In (D) and (E), shading is used to highlight the resonance, which becomes a BIC at ϕ = 0. For (D) and (E), θ = 15° (which corresponds to *k_x_* = 0.19π/*a*), and the purple squares indicate the frequency of the BIC calculated for a slab surrounded by an infinite woodpile environment. Slices along the red dashed lines are depicted in (**F**), where we can see the resonance features in the transmission for ϕ = 15° and ϕ = 7.5°, which vanish at the BIC. Blue arrows are a visual guide to the resonance in question.

We can experimentally observe this line of symmetry-protected BICs using only a single layer of photonic crystal environment. The entire system is fabricated by direct laser writing on top of a glass substrate using two-photon polymerization of a low-index photoresist, ε = 2.34, shown in Fig. 3A (for more details, see Materials and Methods). The rods comprising the rectangular woodpile have a cross section with width *a*/2 and height 1.4*a*, and each layer of the woodpile is separated vertically in *z* by *a* (layers overlap), where *a* = 1 μm is the lattice constant in *x* and *y*. The slab has height *a*. Simulations show that because of the strong localization of the BIC slab modes, only a single unit cell of the woodpile environment is required on each side of the slab to preserve the exponential localization of the resonance due to symmetry (see the Supplementary Materials). In addition, as the line of BICs in the setup of Fig. 2 is under the light line of free space, we use a period-doubled grating written on the top and bottom of the structure [cyan elements in Fig. 3 (A and B)], for in- and outcoupling. This remaps the line of BICs to be along **Γ**−**X** and thus above the light line of free space (see Fig. 3C). The grating elements have width 0.8*a* and heights 1.4*a* (above) and 4*a* (below) (see Materials and Methods). Together, the combined effects of the truncated environment and grating yield a *Q* factor of the confined resonance in excess of 10^6^ (see the Supplementary Materials). Transmission spectra are taken with an FTIR spectrometer by tilting the samples by the angle θ about the *y* axis, while sweeping through the angle ϕ that rotates the sample about the *x* axis (see Fig. 3B). In this measurement scheme, *k_x_* = ω/*c* sin (θ), *k_y_* = ω/*c* cos (θ) sin (ϕ), and the **Γ**−**X** line corresponds to ϕ = 0.

The resonance of the slab that becomes a symmetry-protected BIC at ϕ = 0 can be identified in the angle-resolved transmission spectrum as a concave-up series of avoided crossings for ∣ϕ∣> 0, whose linewidth approaches zero as ∣ϕ∣ → 0. This process is shown in simulations of a substrate-free system in Fig. 3D, where the linewidth of the resonance becomes too narrow to be resolved at this scale for ∣ϕ∣ ≤ 3°. Figure 3E shows a comparison of the experimental observation with the simulation of the complete fabricated structure where, again, the resonance becomes too narrow to be resolved for ∣ϕ∣ ≤ 6°. In the simulation results shown in Fig. 3E, only the specular transmission has been retained, and this channel has been averaged over θ ± 2° and ϕ ± 0.3° to mimic the behavior of the spot shape and pinhole used in the experimental measurements (see Materials and Methods). The vanishing linewidth can also be seen in a series of slices of the experimental data (Fig. 3F). The discrepancies seen between the experiment and the simulation in Fig. 3E are likely due to the difficulty in determining the exact structure parameters using scanning electron microscopy at the edge of the device. In addition, the resonance at ϕ = 0 can be revealed in our experimental observations by purposefully breaking the symmetry of the structure that protects the BIC, reducing its *Q* factor to ~10^2^, shown in fig. S6. Together, these experimental measurements show that this system has a symmetry-protected BIC because of the presence of the surrounding woodpile photonic crystal environment.

**Fig. 4.**
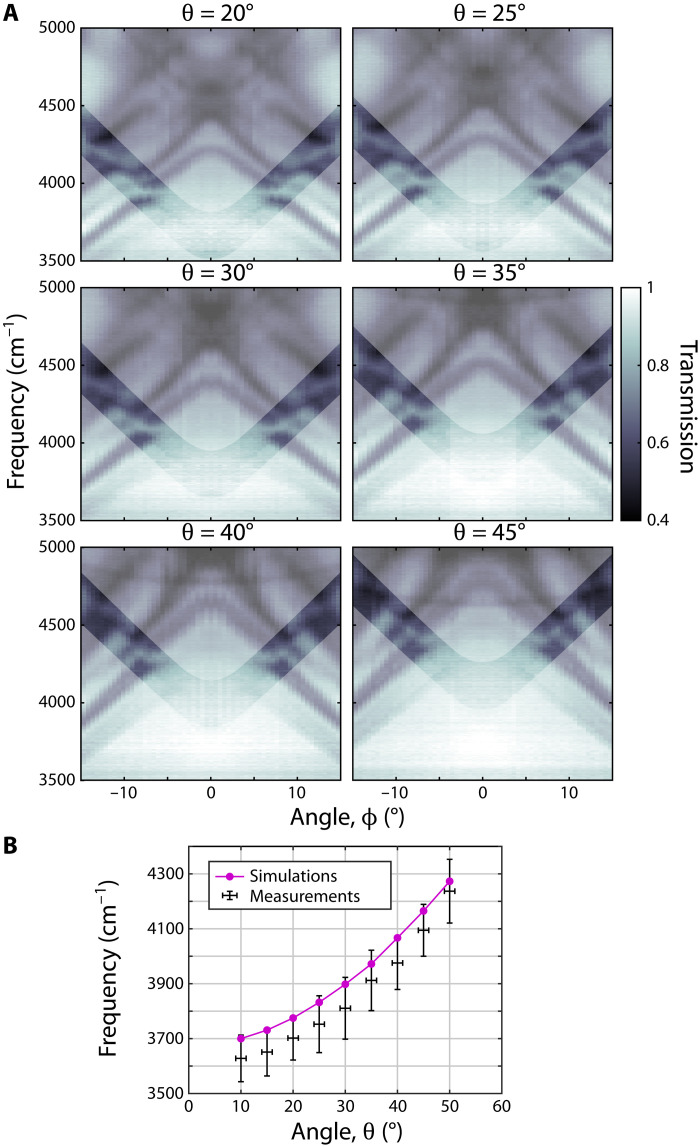
Line of symmetry-protected BICs. (**A**) Experimentally observed transmission spectra of the BIC structure as ϕ is varied and for different positions along **Γ**−**X**, given by θ. Shading is used to highlight the resonance, which vanishes around ϕ = 0. (**B**) BIC frequencies as extracted from the measurements (black) and the simulations (magenta) versus θ. The plotted values of increasing θ correspond to *k_x_* = (π/*a*)[0.13,0.19,0.26,0.32,0.39,0.46,0.52,0.59,0.66].

Last, to demonstrate that our experimental system exhibits a line of symmetry-protected BICs, we repeat the experiment for different values of θ, which, for ϕ = 0, correspond to different wave vectors in the Brillouin zone along the **Γ**−**X** line. As is shown in Fig. 4A, in all cases, the resonance is clearly seen for large values of ∣ϕ∣ but vanishes as ∣ϕ∣→ 0. We estimate the frequencies of the BICs in these measurements (see Materials and Methods) and find excellent agreement with the frequencies obtained by simulations with an average error between the two of Δω/ω = 1.84% (Fig. 4B).

## DISCUSSION

In conclusion, using two-photon polymerization–based 3D microprinting of a photonic device, we have experimentally demonstrated that 3D photonic crystal environments can be used to create symmetry bandgaps and to realize symmetry-protected BICs away from normal incidence, which we analytically prove is impossible with homogeneous, isotropic environments. This greatly expands the design space and the achievable properties of BIC-based devices. Moreover, we have shown not only that symmetry bandgaps can be realized in low-index systems but also that the benefits of environmental design require only a single layer of the environment on both sides of the system. These ideas have immediate ramifications in enabling multifrequency and multi–wave vector applications in technologies using BICs, such as sensors (*25*–*27*) and high-power lasers (*28*).

## MATERIALS AND METHODS

### Fabrication

Samples are fabricated in the IP-Dip resist [for refractive index in the infrared, see (*48*)] using a Nanoscribe Professional GT at a scan speed of 20 mm/s and a laser power of 60% (which corresponds to approximately 33 mW on the entrance lens of the objective). The structures were printed onto Menzel coverslips (borosilicate glass) coated with approximately 13 nm of Al_2_O_3_ to facilitate interface finding in the dip-in configuration. The coverslips have a transmission of greater than 75% for all wavelengths used in our measurements. After printing, the sample is developed for 10 min in PGMEA (propylene glycol monomethyl ether acetate) and 10 min in isopropanol, subsequently. In the last step, the sample is blow-dried in a stream of nitrogen. The complete footprint of the structure is approximately 1 mm^2^. To achieve such a large footprint within reasonable writing time, we stitch the structure out of 4 × 4 angled blocks using stage stitching for larger travel distance. Inside each block and layer, we apply piezo-stitching in combination with galvo scanning for reduced vignetting and more precise positioning. Alignment of stage, piezo, and galvo axes is ensured, using the transformation implemented in NanoWrite. Structural parameters are determined via scanning electron microscopy.

For the structure with unbroken symmetry, they are as follows: lattice constant *a* = 1.01(1) μm; rod width in *x*, *r_x_* = 0.50(4) μm; rod width in *y*, *r_y_* = 0.56(6) μm; and width of the grating *r*_g_ = 0.78(3) μm. The layer heights in *z* from top to bottom are *h*_top,grating_ = 1.44(4) μm, *h*_top,rods,*y*_ = 1.33(2) μm, *h*_top,rods,*x*_ = 1.37(2) μm, slab height *h*_s_ = 1.01(5) μm, *h*_bottom,rods,*x*_ = 1.45(5) μm, and *h*_bottom,rods,*y*_ = 1.45(2) μm. The bottom grating differs in height across the footprint of the structure because of a slight tilt of the substrate during the fabrication and lies between 4.0 and 5.5 μm. The height of the lower grating was chosen such that any (unpredictable) tilt of the substrate during fabrication would not result in a complete vanishing of the grating anywhere across the structure.

For the symmetry-broken structure (shown in the Supplementary Materials), the parameters are as follows: *a* = 1.00(2) μm, *r_x_* = 0.49(2) μm, *r_y_* = 0.59(1) μm, and *r*_g_ = 0.68(1) μm. The layer heights in *z* from top to bottom are *h*_top,grating_ = 1.38(7) μm, *h*_top,rods,*y*_ = 0.80(6) μm, *h*_top,rods,*x*_ = 1.35(4) μm, slab height *h*_s_ = 0.82(4) μm, *h*_bottom,rods,*x*_ = 1.23(5) μm, and *h*_bottom,rods,*y*_ = 1.15(4) μm. The bottom grating differs in height across the footprint of the structure and lies between 1.1 and 3.9 μm.

### Measurement

To measure the spectra of the samples, we use the HYPERION 3000 microscope attached to a Bruker VERTEX v70 FTIR. The spectra are taken with a nitrogen-cooled MCT (mercury-cadmium-telluride) detector and a halogen lamp in transmission mode. To increase *k*-space resolution, the lower 15× Cassegrain objective is covered except for a pinhole of 1 mm in diameter, such that we obtain a nearly collimated beam. We fix the value of *k_x_* (angle θ to the *x* axis) by first tilting the sample by θ around the *y* axis and then scan through *k_y_* by tilting the sample with respect to the beam around the *x* axis. This is done by tilting the sample holder in steps of approximately 0.5°. Because we do not know the exact position of perpendicular incidence of the beam relative to the sample from the setup, we determine *k_y_* = 0 from the symmetry of the measured angle-resolved transmission spectra. All spectra are referenced to the transmission of the used substrates. For each spectrum taken, we average 64 measurements with an FTIR resolution set to 4 cm^−1^ in wave number. The small dip in transmission around 3500 cm^−1^, constant across angles, is due to the absorption in the IP-Dip resist (*48*).

It is difficult to extract the exact frequencies of the BICs from the experiments because the Fano feature of the slab resonance vanishes around the position of the BICs (i.e., the transmission becomes unity). Therefore, the frequencies of the BICs for a given θ shown in Fig. 4B were estimated the following way: For each θ, we set the upper limit of the BIC frequency to be the horizontal connection between the frequencies for which the resonance was just barely visible. To obtain the lower limit, we drew a linear continuation of the resonance at both sides of ϕ = 0 and extracted the frequency at their intersection at ϕ = 0. The plotted points in Fig. 4B are then the average of these two frequency limits for each respective θ, while their difference gives the vertical error bar.

### Numerical methods

The numerical simulations shown here were performed using three different software packages, MIT (Massachusetts Institute of Technology) Electromagnetic Equation Propagation (MEEP) (*49*), MIT Photonic Bands (MPB) (*50*), and Stanford Stratified Structure Solver (S^4^) (*51*). The projected-in-*k_z_* band structures of the rectangular woodpile environment shown in Fig. 2B, as well as the modal profiles of the environment in Fig. 2C, were calculated using MPB. Calculations of the *Q* factor and field profiles of the resonances of the slab, such as the purple line in Fig. 2B, the resonance profile in Fig. 2E, and the properties of the symmetry-detuned system in Fig. 2F, were performed using MEEP. Last, numerical simulations of the transmission spectrum in Fig. 3 (D and E) were performed using S^4^. Note that the purple squares in Fig. 3 (D to F) and the purple data in Fig. 4B were calculated using MEEP.

In simulations of the system with an infinite environment, i.e., those in Fig. 2 and fig. S4, the environmental layers above and below the slab were all taken to have the dimensions shown in Fig. 2A, and the dielectric of all of the photoresist structures is assumed to be ε = 2.34. For fig. S4, the top and bottom period-doubled gratings are taken to both have height 1.4*a* and width 0.79*a*. However, simulations of the finite system shown in Figs. 3 and 4 all use the scanning electron microscope–measured dimensions of the system given in the “Fabrication” section and assume that all the photoresist structures have dielectric ε = 2.34 + 0.005*i*, to approximate the effects of surface roughness. Note that, because of the fabrication process, these layers all overlap. Hence, the heights quoted are for the height of each rod from the bottom of that rod to the top, but the vertical separation between the adjacent layers of rods is approximated to *a*. In other words, the rectangular woodpile unit cell has dimensions *a* by *a* by 2*a*. For the S^4^ simulations in Fig. 3E, a glass substrate, ε = 2.25, with a height of 170*a* was included and the samples are measured (in the experiment and thus also in the simulations) “bottom-up,” i.e., the incident light first transmits or reflects off of the glass substrate, then the “bottom” period-doubled grating, and so on, until finally escaping out the other side from the “top” grating. Moreover, for S^4^ simulations in Fig. 3E, the high-frequency Fabry-Perot resonances from the thick glass substrate were filtered out using Fourier analysis.

## Supplementary Materials

### This PDF file includes:

Supplementary Text Figs. S1 to S6 References
