# Supplementary material for: Observation of bound states in the continuum embedded in symmetry bandgaps
Source: Sci Adv. 2021 Dec 22;7(52):eabk1117. doi: 10.1126/sciadv.abk1117 (PMC8694597; doi:10.1126/sciadv.abk1117)
Supplement: Supplementary file 1 — Supplementary Text Figs. S1 to S6 References [file sciadv.abk1117_sm.pdf]

Science Advances
AAAS

Supplementary Materials for

Observation of bound states in the continuum embedded in symmetry bandgaps

Alexander Cerjan*, Christina Jörg*, Sachin Vaidya, Shyam Augustine, Wladimir A. Benalcazar, Chia Wei Hsu, Georg von Freymann, Mikael C. Rechtsman

*Corresponding author. Email: awcerja@sandia.gov (A.C.); christinaijoerg@gmail.com (C.J.)

Published 22 December 2021, *Sci. Adv.* **7**, eabk1117 (2021)
DOI: 10.1126/sciadv.abk1117

This PDF file includes:

Supplementary Text
Figs. S1 to S6
References

SI. BRAGG-DIFFRACTION ORDERS

A system consisting of a photonic crystal slab of finite thickness embedded in a homogeneous environment, such as vacuum, possesses discrete translational symmetry in the two-dimensional plane of the photonic crystal,

$$\epsilon(\mathbf{r}) = \epsilon(\mathbf{r} + l_1\mathbf{a}_1 + l_2\mathbf{a}_2), \tag{S1}$$

where $\mathbf{a}_{1,2}$ are the lattice vectors of the photonic crystal slab and $l_{1,2} \in \mathbb{Z}$. As any resonance of the photonic crystal slab must satisfy Maxwell's wave equation, and Maxwell's wave equation commutes with all of the possible discrete translational symmetry operations in Eq. (S1), the resonances of the slab can be constructed to be eigenfunctions of both the wave equation and the symmetry operators simultaneously. This means that the resonances of the slab can be categorized based on their in-plane wavevector, $\mathbf{k}_\parallel = (k_x, k_y)$, which are the eigenvalues of the discrete translation operators and are uniquely defined up to the reciprocal lattice vectors, $\mathbf{b}_i \cdot \mathbf{a}_j = 2\pi\delta_{i,j}$. In other words, resonances of the slab at $\mathbf{k}_\parallel$ are equivalent to, and have the same frequencies as, those found at $\mathbf{k}_\parallel + \sum_{i=1,2} m_i\mathbf{b}_i$, where $m_i \in \mathbb{Z}$.

However, for a radiative channel to exist in the surrounding homogeneous environment, where there is continuous translational symmetry, the propagating light must satisfy the dispersion relation in that medium,

$$\frac{n^2\omega^2}{c^2} = k_x^2 + k_y^2 + k_z^2, \tag{S2}$$

where n is the refractive index of the environment and ω is the light's frequency. In particular, this means that for light in the slab at a given frequency to radiate, it must possess a frequency and in-plane wavevector that yield a real out-of-plane momentum, k_z,

$$k_z = \sqrt{\frac{n^2\omega^2}{c^2} - \left(\mathbf{k}_\parallel + \sum_{i=1,2} m_i\mathbf{b}_i\right)^2}. \tag{S3}$$

Note, that although resonances of the photonic crystal slab at $\mathbf{k}_\parallel + \sum_{i=1,2} m_i\mathbf{b}_i$ are equivalent, these correspond to inequivalent radiative channels in free-space. Equation (S3) thus defines a series of frequency cutoffs for how many radiative channels are available for a given resonance to radiate to.

- If $n^2\omega^2/c^2 < \mathbf{k}_\parallel^2$, i.e. $m_i = 0$, the resonance is below the light line and is a bound state, perfectly confined by total internal reflection.
- If $n^2\omega^2/c^2 > \mathbf{k}_\parallel^2$, but $n^2\omega^2/c^2 < (\mathbf{k}_\parallel + \sum_i m_i\mathbf{b}_i)^2$ for $m_i \neq 0$, only a pair of radiative channels are available, both have the same k_z, but have orthogonal polarizations. In this case, the resonance of the photonic crystal slab is said to be below the first Bragg-diffraction limit.
- If $n^2\omega^2/c^2 > (\mathbf{k}_\parallel + \sum_i m_i\mathbf{b}_i)^2$ for some set of $(m_1, m_2) \neq (0, 0)$, there are additional pairs of radiative channels available with distinct wavevectors and the resonance is above the first Bragg-diffraction limit, though it is below some subsequent Bragg-diffraction limit.

These different regions are schematically illustrated in Fig. 1 in the main text.

SII. PROOF – NO SYMMETRY-PROTECTED BICS ABOVE THE BRAGG-DIFFRACTION LIMIT

In this section, we will provide a detailed proof of the claim that:

> **Theorem** *For a two-dimensional photonic system with finite thickness embedded in a homogeneous three-dimensional environment, symmetry-protected BICs can only be found below the first Bragg-diffraction limit at* $\mathbf{\Gamma}$.

The question of whether or not a symmetry-protected BIC can be found in a photonic crystal slab amounts to the question, 'For every choice of $\mathbf{k}_\parallel$, do the available radiative channels span all of the possible symmetries of the slab's resonances at that wavevector?' Or in other words, 'At every $\mathbf{k}_\parallel$, do the radiative channels exhaust the irreducible representations of the little group, $\mathscr{G}(\mathbf{k}_\parallel)$?' If there are irreducible representations present in $\mathscr{G}(\mathbf{k}_\parallel)$ which are unavailable in the radiative channels, then any slab resonance obeying that combination of symmetries

will necessarily be a BIC at that $\mathbf{k}_{\parallel}$. Thus, to prove that symmetry-protected BICs cannot exist above the Bragg-diffraction limit, we must show that above this limit the radiative channels always span the irreducible representations of $\mathscr{G}(\mathbf{k}_{\parallel})$, regardless of the specific symmetries of the system.

In two dimensions, there are 17 possible space groups (i.e. the 'wallpaper groups') which consist of different combinations of three classes of symmetry operations, rotations, reflections, and glides, which form the point group of the structure, along with the translational symmetry of the system. As such, the in-plane symmetry of the photonic crystal slab must be one of these 17 space groups. Here, we are omitting any consideration of the symmetry of the system in the perpendicular direction for two reasons. First, many photonic crystal slabs and metasurfaces are constructed on a substrate with a different refractive index from the material above the structure, and such systems are not symmetric in the perpendicular direction. Second, even for suspended structures, or those which use an index-matching solution to restore reflection symmetry about the xy-plane (σ_z), it is not possible to use this out-of-plane reflection symmetry to construct a BIC in the slab. This is because the total possible symmetries of the photonic crystal slab of a σ_z symmetric system are simply the direct product of this single out-of-plane symmetry with the in-plane symmetries, but the available radiative channels above and below the slab are degenerate, so linear combinations of these degenerate channels span all of the available out-of-plane symmetries of the slab.

Here, we adopt the notation from Ref. 52 from the main text, in which we represent symmetry operations in the form $\{\beta|\mathbf{B}\}$, such that

$$\{\beta|\mathbf{B}\}\mathbf{r} = \beta\mathbf{r} + \mathbf{B}, \tag{S4}$$

where β is a 3×3 matrix (or for two-dimensional systems 2×2 matrix) which rotates or reflects $\mathbf{r}$, while $\mathbf{B}$ denotes a possible translation. When operating on a function, this acts as

$$\{\beta|\mathbf{B}\}\psi(\mathbf{r}) = \psi(\beta^{-1}\mathbf{r} - \beta^{-1}\mathbf{B}). \tag{S5}$$

In this nomenclature, the identity operation is $\{\varepsilon|0\}$, and we use $\{C_n|0\}$ to denote counter-clockwise rotation by $2\pi/n$ about the z-axis, $\{\sigma|0\}$ to denote a reflection about a specified plane, and $\{\sigma|\boldsymbol{\tau} \neq 0\}$ to denote a glide operation.

Finally, we remind the reader that the slab resonances at a given $\mathbf{k}_{\parallel}$ may have lower symmetry than the point group of the photonic crystal slab itself. The symmetries, $\{\beta|\mathbf{B}\}$, which the slab resonances at $\mathbf{k}_{\parallel}$ must obey (i.e., those symmetry operations which constitute the little group $\mathscr{G}(\mathbf{k}_{\parallel})$) are those for which

$$\beta\mathbf{k}_{\parallel} = \mathbf{k}_{\parallel} + \sum_{i=1,2} m_i \mathbf{b}_i, \tag{S6}$$

for some choice of integers $m_{1,2}$, such that $\beta\mathbf{k}_{\parallel}$ is equivalent to $\mathbf{k}_{\parallel}$ up to a reciprocal lattice vector. This equivalence is denoted as

$$\beta\mathbf{k}_{\parallel} \doteq \mathbf{k}_{\parallel}. \tag{S7}$$

A. Basis for radiative channels

To study the available symmetries of the radiative channels of the environment propagating away from the slab in the $+z$ direction, we choose a basis of these plane wave channels based on their three distinguishing characteristics, $|n_z, n_d, s/p\rangle$, which correspond to:

- $n_z \in [0, N_z - 1]$ is the Bragg-diffraction order the channel is in. This value is ordered in decreasing k_z, and is indexed from 0, such that $n_z = 0$ is below the first Bragg-diffraction limit and corresponds to 'specular radiation' with $m_{1,2} = 0$ in Eq. (S3). The upper bound N_z is the number of unique values of real k_z for all $m_{1,2}$ given ω and $\mathbf{k}_{\parallel}$ of the slab resonance.
  Note – for $\mathbf{k}_{\parallel}$ along the boundary of the Brillouin zone, there are no frequencies which are both above the light line and below the first Bragg-diffraction limit, as k_z for $m_{1,2} = 0$ is always degenerate with k_z for $m_{1,2}$ corresponding to the neighboring Brillouin zone, see Fig. 1 in the main text. In this case, there is no specular radiation, and $n_z \in [1, N_z - 1]$.

- $n_d \in [0, N_d - 1]$ labels the in-plane wavevector of the channel in the environment, $\mathbf{k}_{\parallel} + \sum_{i=1,2} m_i \mathbf{b}_i$. The total number of possible in-plane wavevectors for a given Bragg-diffraction order, N_d, is equal to the number of unique pairs (m_1, m_2) which yield the same k_z via Eq. (S3).
  Note – For $\mathbf{k}_{\parallel} \neq \mathbf{\Gamma}$, or $\mathbf{k}_{\parallel} = \mathbf{\Gamma}$ and $n_z \neq 0$, N_d must be an integer multiple of the number of rotational symmetry operators which leave $\mathbf{k}_{\parallel}$ invariant.

Proof of note – This follows from the definition of the little group of $\mathbf{k}_\parallel$. If $\{C_n|0\} \in \mathscr{G}(\mathbf{k}_\parallel)$, then $C_n\mathbf{k}_\parallel \doteq \mathbf{k}_\parallel$. But given the stated assumptions ($\mathbf{k}_\parallel \neq \mathbf{\Gamma}$, or $\mathbf{k}_\parallel = \mathbf{\Gamma}$ and $n_z \neq 0$), then $C_n\mathbf{k}_\parallel \neq \mathbf{k}_\parallel$ except for $C_n = C_1$, so there must be some other pair (m_1, m_2) which yields an equivalent in-plane wavevector with the same k_z, as rotation about the z axis cannot change the momentum in the z direction. As different rotation operations will result in different in-plane wavevectors, these new pairs generated (m_1, m_2) from the rotation operations $C_n \neq C_{n'}$ must be unique.

- s/p denotes the polarization of the channel, where s corresponds to the radiation channel whose electric field vector is parallel to the photonic crystal slab. For $n_z = 0$ at $\mathbf{k}_\parallel = \mathbf{\Gamma}$, this choice is arbitrary, but the two polarizations are chosen to be orthogonal.

An example of this choice of basis for specular radiation, $n_z = 0$ and the first Bragg-diffraction order, $n_z = 1$, is shown in Fig. S1 for a photonic crystal slab whose unit cell is C_{4v} symmetric at $\mathbf{k}_\parallel = \mathbf{\Gamma}$.

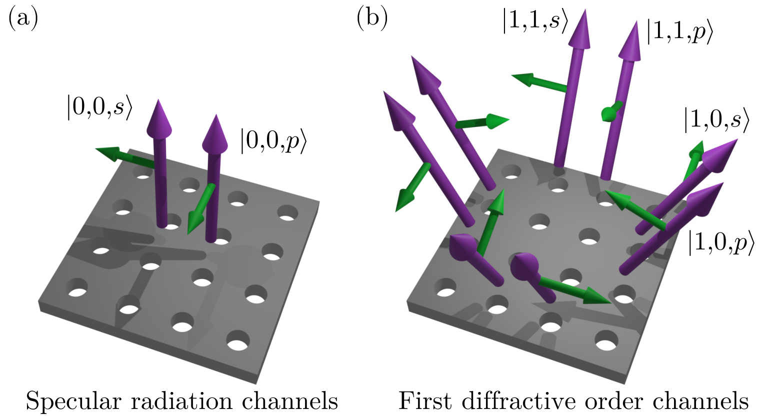

FIG. S1. **Choice of basis for the radiative channels.** Here, the basis, $|n_z, n_d, s/p\rangle$, is shown for a free-space environment at $\mathbf{k}_\parallel = \mathbf{\Gamma}$ that surrounds a photonic crystal slab whose unit cell is C_{4v} symmetric. (a) Members of the basis corresponding to specular radiation, $n_z = 0$. Here, the purple arrow denotes the wavevector of the radiative channel, $\mathbf{k}$, and the green arrow denotes the polarization of the electric field. (b) Members of the basis corresponding to the first Bragg-diffraction order, $n_z = 1$. The four unique choices of (m_1, m_2) which constitute this set of in-plane wavevectors correspond to $(\pm 1, 0)$ and $(0, \pm 1)$.

Using this basis, it is easy to verify that the representation matrices, $D(\{\beta|\mathbf{B}\})$, of the symmetry operations $\{\beta|\mathbf{B}\} \in \mathscr{G}(\mathbf{k}_\parallel)$ are block-diagonal,

$$D(\{\beta|\mathbf{B}\}) = \begin{pmatrix} D^{(n_z=0)}(\{\beta|\mathbf{B}\}) & 0 & \cdots \\ 0 & D^{(n_z=1)}(\{\beta|\mathbf{B}\}) & \cdots \\ \vdots & \vdots & \ddots \end{pmatrix}, \tag{S8}$$

in which each block corresponds to the representation of that symmetry operation for a different Bragg-diffraction order of the system. This property is a consequence of the fact that none of the in-plane symmetry operations can change the out-of-plane wavevector. As such, we can treat the representations of the different Bragg-diffraction orders separately, and the total symmetries spanned by all of the Bragg-diffraction orders at a given $\mathbf{k}_\parallel$ will be the direct sum of those symmetries spanned by each order.

B. Structure of the rotation representation matrices

For any non-trivial rotation operation, $\{C_{n\neq 1}|0\}$, which leaves $C_{n\neq 1}\mathbf{k}_\parallel \doteq \mathbf{k}_\parallel$, the effect of this operation on the chosen basis only results in non-diagonal elements of the representation of this rotation, $D^{(n_z\geq 1)}(\{C_{n\neq 1}|0\})$, for any non-zero Bragg-diffraction order. This is a consequence of the fact that for any such radiative channel, $|n_z \geq 1, n_d, s/p\rangle$, a rotation operation necessarily changes the in-plane component of its wavevector, changing n_d. An example of the action of these rotations on the chosen basis is shown in Fig. S2.

As such, the character, $\chi(\{\beta|\mathbf{B}\}) = \mathrm{Tr}[D(\{\beta|\mathbf{B}\})]$, of any non-trivial rotation for any non-zero Bragg-diffraction order is zero,

$$\chi^{(n_z\geq 1)}(\{C_{n\neq 1}|0\}) = 0. \tag{S9}$$

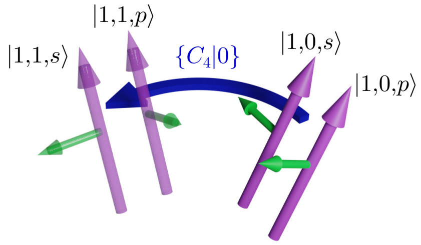

FIG. S2. **Effects of rotation operations.** Example of a $\{C_4|0\}$ rotation operation applied to some basis elements of the system from Fig. S1. For this system, $D(\{C_4|0\})|1,0,s/p\rangle = |1,1,s/p\rangle$.

C. Structure of the reflection representation matrices

There are two possible scenarios that can arise when a reflection operation, $\{\sigma|0\}$, which leaves $\sigma\mathbf{k}_{\|} \doteq \mathbf{k}_{\|}$, acts on one of the radiative channel basis functions. If the basis function's wavevector, $\mathbf{k}$, does not lie in the reflection plane, then the reflection operation maps that basis function to a different member of the radiative basis. However, if $\mathbf{k}$ lies in the reflection plane then $D(\{\sigma|0\})|n_z,n_d,s/p\rangle \propto |n_z,n_d,s/p\rangle$. These two possibilities are shown in Fig. S3.

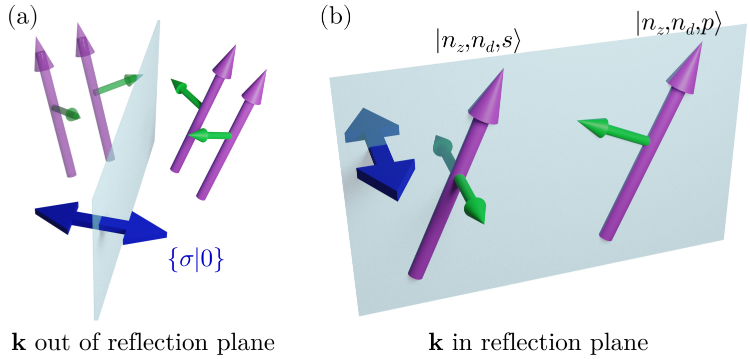

FIG. S3. **Effects of reflection operations.** Examples of reflection operations acting on radiative channel basis functions where the channel's wavevector, $\mathbf{k}$, either lies out of the reflection plane (a), or in the reflection plane (b).

Due to the environment being homogeneous, the frequencies and out-of-plane wavevectors for s and p polarized light are degenerate. Thus, as can be seen in Fig. S3b, when the wavevector of the radiation channel lies in the reflection plane,

$$D(\{\sigma|0\})|n_z,n_d,s\rangle = -|n_z,n_d,s\rangle, \quad \text{(S10)}$$

$$D(\{\sigma|0\})|n_z,n_d,p\rangle = +|n_z,n_d,p\rangle. \quad \text{(S11)}$$

As such, regardless of the particular reflection operation, while the individual diagonal elements of $D(\{\sigma|0\})$ may be non-zero, the character is necessarily zero,

$$\chi^{(n_z\geq 1)}(\{\sigma|0\}) = 0. \quad \text{(S12)}$$

as s and p polarized channels must appear in the radiative basis in pairs.

D. Structure of the glide representation matrices

As a reminder, glide operations are the combination of a reflection operation with translation by a vector which cannot be expressed as an integer multiple of lattice vectors, and can be expressed as the product of these two operations, $\{\sigma|\boldsymbol{\tau}\} = \{\varepsilon|\boldsymbol{\tau}\}\{\sigma|0\}$. Given that we already know the action of the reflection operation on the radiation basis from the previous section, we must understand the effect of translation on the radiative basis. As the radiative basis consists of plane wave propagating away from the photonic crystal slab, we know the functional form of these states far away from the crystal slab,

$$|n_z,n_d,s/p\rangle = \mathbf{e}_{s/p}e^{i\mathbf{k}\cdot\mathbf{r}-i\omega t}, \quad \text{(S13)}$$

where $\mathbf{e}_{s/p}$ is a unit vector pointing in the direction of the polarization of the electric field. Thus, using Eq. (S5), we can calculate the effect of translation on this basis element,

$$D(\{\varepsilon|\boldsymbol{\tau}\})|n_z, n_d, s/p\rangle = \mathbf{e}_{s/p} e^{i\mathbf{k}\cdot(\mathbf{r}-\boldsymbol{\tau})-i\omega t},$$
$$= e^{-i\mathbf{k}\cdot\boldsymbol{\tau}}|n_z, n_d, s/p\rangle. \quad \text{(S14)}$$

As can be seen, this translation effects the two polarizations of radiation channels identically, and thus again, while the individual diagonal elements of $D(\{\sigma|\boldsymbol{\tau}\})$ may be non-zero, the character is necessarily zero,

$$\chi^{(n_z\geq 1)}(\{\sigma|\boldsymbol{\tau}\}) = 0, \quad \text{(S15)}$$

regardless of the specifics of the particular glide operation.

E. Completing the proof

Over the previous subsections, we have proven that the characters for all of the representations of the symmetry operations, $\{\beta|\mathbf{B}\}$, for which $\beta\mathbf{k}_\parallel \doteq \mathbf{k}_\parallel$ are zero, except for that of the identity operation,

$$\chi^{(n_z\geq 1)}(\{\varepsilon|0\}) = 2N_d, \quad \text{(S16)}$$
$$\chi^{(n_z\geq 1)}(\{\beta|\mathbf{B}\}) = 0, \qquad \text{otherwise.} \quad \text{(S17)}$$

Thus, upon using the orthogonality of characters of irreducible representations (see Ref. 52) to decompose the representations of the non-zero Bragg-diffraction orders of the radiative channels, we find that

$$\begin{aligned} q_{(\alpha)} &= \frac{1}{g}\sum_{\{\beta|\mathbf{B}\}\in\mathscr{G}(\mathbf{k}_\parallel)} \chi_{(\alpha)}(\{\beta|\mathbf{B}\})^*\chi^{(n_z\geq 1)}(\{\beta|\mathbf{B}\}) \\ &= \frac{1}{g}\chi_{(\alpha)}(\{\varepsilon|0\})^*\chi^{(n_z\geq 1)}(\{\varepsilon|0\}) \\ &= \frac{2N_d}{g}\chi_{(\alpha)}(\{\varepsilon|0\})^*. \end{aligned} \quad \text{(S18)}$$

Here, α denotes an irreducible representation of $\mathscr{G}(\mathbf{k}_\parallel)$, $\chi_{(\alpha)}(\{\beta|\mathbf{B}\})$ is the character of that irreducible representation for $\{\beta|\mathbf{B}\} \in \mathscr{G}(\mathbf{k}_\parallel)$, g is the number of elements in $\mathscr{G}(\mathbf{k}_\parallel)$, and $q_{(\alpha)}$ is the number of times that irreducible representation appears in the representation of the environment's radiative channels. As the character of the identity operation for every irreducible representation is its dimension, $\chi_{(\alpha)}(\{\varepsilon|0\})^* \geq 1$, and so

$$q_{(\alpha)} > 0 \quad \forall\alpha. \quad \text{(S19)}$$

Thus, for any non-zero Bragg-diffraction order of a homogeneous radiative environment surrounding a photonic crystal slab, the radiative channels completely span the possible symmetries of the modes of the photonic crystal slab. As such, one cannot find symmetry-protected bound states in the continuum above the Bragg-diffraction limit. In particular, this means that there cannot be symmetry-protected BICs at the high-symmetry points along the boundary of the Brillouin zone, as the boundary of the Brillouin zone is always above the first Bragg-diffraction limit.

SIII. PROOF – NO SYMMETRY-PROTECTED BICS BELOW BRAGG-DIFFRACTION LIMIT EXCEPT AT $\mathbf{k}_\parallel = \boldsymbol{\Gamma}$

For the 17 possible space groups of two-dimensional systems, the only possible symmetry classes which can be found on the interior of the Brillouin zone away from $\mathbf{k}_\parallel = \boldsymbol{\Gamma}$ is even/odd symmetry with respect to a reflection (or glide) operation. In other words, except for $\boldsymbol{\Gamma}$ (and the boundary of the Brillouin zone which is above the first Bragg-diffraction limit), the maximum number of elements which can be found in $\mathscr{G}(\mathbf{k}_\parallel)$ is 2, along high-symmetry lines for which $\mathbf{k}_\parallel$ lies in the plane of a reflection or glide symmetry. In this case, the point group of $\mathscr{G}(\mathbf{k}_\parallel)$ is C_{1h}, and resonances of the photonic crystal slab at this wavevector will either be even or odd with respect to this reflection or glide symmetry operation. However, one can quickly verify that the specular radiation channels in the surrounding environment (those with $n_z = 0$), span the two possible symmetries of the slab's resonances, as the s polarized channel will be odd about the reflection operation, while the p polarized channel will be even, see Fig. S3b.

Thus, below the first Bragg-diffraction limit for $\mathbf{k}_\parallel \neq \boldsymbol{\Gamma}$, there are no symmetry-protected BICs, as the specular radiation channels exhaust the possible symmetries of the resonances of the slab.

SIV. TRUNCATION OF THE ENVIRONMENT

A. Truncation in z

In this section, we numerically justify the truncation of the infinite environment to an environmental layer a single unit cell thick on each side of the slab. In the absence of an infinite rectangular woodpile environment surrounding the slab, and with the addition of the period-doubled grating, the symmetry-protected resonance of the slab can evanescently couple to the grating layers and radiate into the surrounding air, as the resonance along the $\mathbf{\Gamma}$–$\mathbf{X}$ line is not a symmetry-protected BIC when the radiative environment is free space. In this picture, the layers of the rectangular woodpile act as a barrier without any states the slab resonance can couple to, which increases the distance between the slab and the grating. This lack of states with the correct symmetry in the woodpile photonic crystal means that the coupling between the slab resonance and the grating is strictly evanescent, and thus decreases exponentially with distance.

Numerically, we can confirm that the resonance frequency and modal profile remain essentially unchanged when the environment is truncated from being infinite, to being comprised of 5 layers of photonic crystal on each side of the slab, see Fig. S4. Moreover, the frequency and modal profile remain nearly fixed as the environment is reduced to being only a single layer of the photonic crystal on each side, with this state still possessing a Q-factor $> 10^6$. However, both of these features dramatically change if the environment is removed entirely. Then, the slab resonance can couple directly to the grating, changing its frequency and modal profile, and significantly reducing its Q-factor.

Thus, the truncation of the environment to a single unit cell thick on either side of the slab is justified, and does not alter the essential physics of the system. In contrast, completely removing the environment would significantly change the properties of the slab resonances.

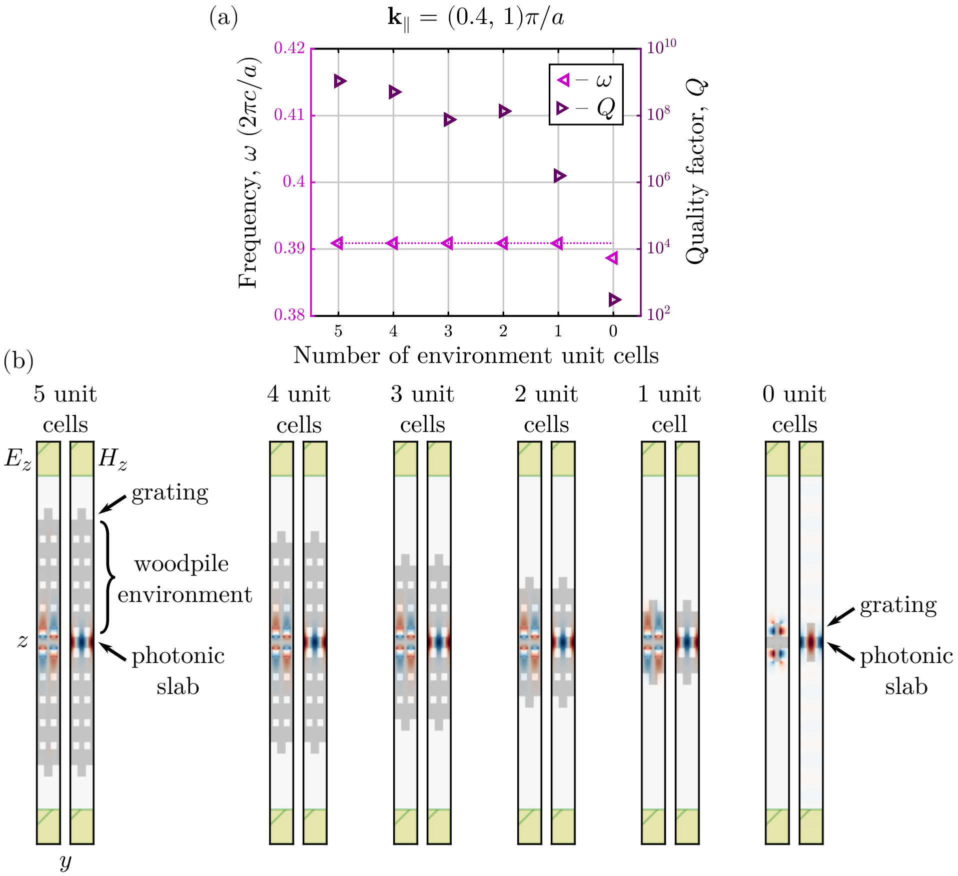

FIG. S4. **Truncation in the out-of-plane direction.** (a) Frequencies (left triangles) and quality factors (right triangles) of the photonic slab resonance as the environment is truncated from from being 5 unit cells thick on each side to vanishing. This system is the same as the one considered in Fig. 3 of the main text, and includes the period-doubled grating. (b) Plot of the E_z and H_z field profiles in the yz-plane through this truncation process. The gray regions denote the dielectric regions, and the yellow boundaries of these plots denote the regions of the perfectly-matched layers absorbing boundary condition.

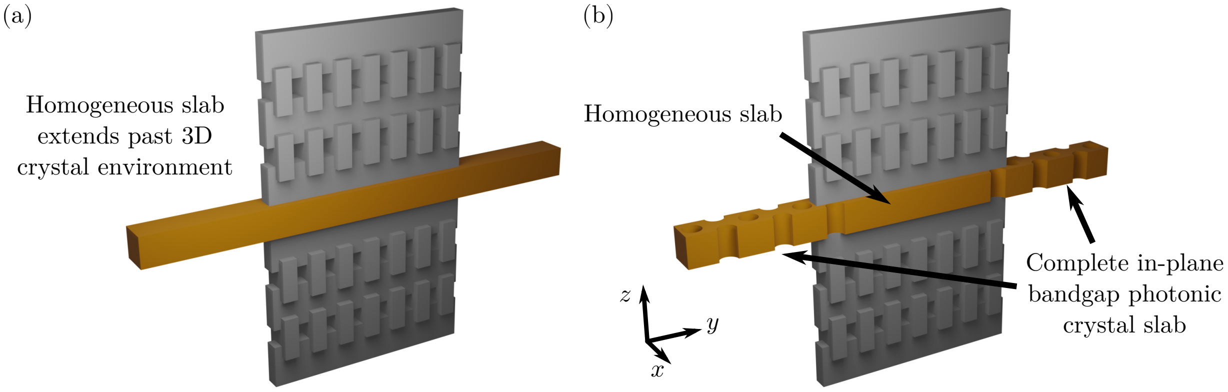

FIG. S5. **Truncation in a direction perpendicular to the slab plane.** These schematics are to be seen as unit cells that are repeated periodically in the x-direction. (a) Homogeneous slab extends beyond the truncated environment. (b) Homogeneous slab is terminated at a photonic crystal slab. The photonic crystal slab is designed to possess a complete in-plane photonic bandgap at the frequency of the symmetry bandgap.

B. Truncation perpendicular to the slab plane

Truncation of the system in a direction perpendicular to the slab plane, such as x or y, can result in a variety of outcomes depending upon the details of the truncation. For this subsection, we will assume that the environment, where it exists, remains infinite in z.

If only the environment is truncated in a direction perpendicular to the slab plane, but the slab itself continues so that there is an area where the slab is not surrounded by the environment (Fig. S5a), this will yield an effective decay rate to the slab states, turning the BICs into finite-Q resonances. Without the environment, the slab states can couple to the surrounding free-space radiative channels, and thus leak. However, the finite-Q resonances will still essentially form a line, as the slab remains infinite in the in-plane directions.

If the environment is truncated in an in-plane direction *and* the slab is also truncated with an in-plane mirror, say by adding an in-plane photonic crystal slab decoration with a complete bandgap where the environment has been removed (Fig. S5b), then the BICs will become finite-Q but still remain high-Q, as the solid slab is still completely surrounded by the environment, while propagation outside of this region is prohibited by the in-plane mirror at the boundary due to the photonic crystal slab. Then:

- If the truncation results in a large region of solid slab surrounded by a environment and the line of BICs exists along the remaining infinite in-plane direction, the line of BICs will be nearly preserved. An example such setup is shown in Fig. S5b, where the system retains discrete translational symmetry in x indefinitely (and thus k_x remains a well-defined wave vector component), but the homogeneous slab is finite in y. Here, the line of BICs previously along **Y**–**M** will remain a line of high-Q resonances if the truncation length in y is sufficiently large such that the standing waves which form in y with the nearly correct symmetry still fall within the original symmetry bandgap of the infinite environment.

- However, if the in-plane truncation results in only a finite width of slab with the environmental cladding and this finite truncation is in the direction of the line of BICs, this will discretize the line of BICs into a set of high-Q resonances at points with spacing $\delta k = 2\pi/L$, where L is the width of the solid slab in the finite direction. This is similar to the effect discussed in Ref. 53 of the main text. An example of such a system is also shown in Fig. S5b, where we now focus on the line of BICs in the non-truncated system along the **X**–**M** high symmetry line. When the system is truncated in y, k_y is no longer a well-defined wave vector component, so the line of BICs will discretize into a set of points that remain high-Q.

SV. BROKEN SYMMETRY EXPERIMENT

To experimentally demonstrate that the BIC is protected by the symmetry of the system, we purposefully break this symmetry in the same manner as is shown in Fig. 2f,g of the main text. As can be seen in Fig. S6, for a system with broken symmetry that is otherwise similar to the system used to observe Figs. 3e and 4 in the main text (see

Materials and Methods section), the resonance now has a finite linewidth at $\phi = 0°$ which can be observed using our experimental methods.

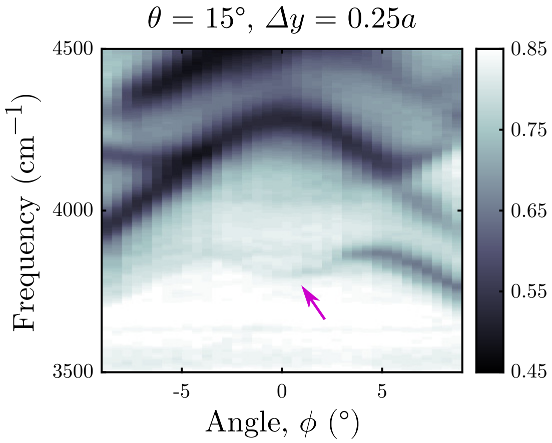

FIG. S6. **Experimentally observed transmission spectrum for a symmetry-broken structure.** Here, we use $\Delta y = 0.25a$ at $\theta = 15°$. See Fig. 2f,g in the main text for the definition of Δy. The purple arrow points to the location of the former-BIC resonance at $\phi = 0°$.

REFERENCES AND NOTES

1. C. W. Hsu, B. Zhen, A. D. Stone, J. D. Joannopoulos, M. Soljačić, Bound states in the continuum. *Nat. Rev. Mater.* **1**, 16048 (2016).

2. H. Friedrich, D. Wintgen, Interfering resonances and bound states in the continuum. *Phys. Rev. A* **32**, 3231–3242 (1985).

3. P. Paddon, J. F. Young, Two-dimensional vector-coupled-mode theory for textured planar waveguides. *Phys. Rev. B* **61**, 2090–2101 (2000).

4. T. Ochiai, K. Sakoda, Dispersion relation and optical transmittance of a hexagonal photonic crystal slab. *Phys. Rev. B* **63**, 125107 (2001).

5. V. Pacradouni, W. J. Mandeville, A. R. Cowan, P. Paddon, J. F. Young, S. R. Johnson, Photonic band structure of dielectric membranes periodically textured in two dimensions. *Phys. Rev. B* **62**, 4204–4207 (2000).

6. S. Fan, J. D. Joannopoulos, Analysis of guided resonances in photonic crystal slabs. *Phys. Rev. B* **65**, 235112 (2002).

7. E. N. Bulgakov, A. F. Sadreev, Bound states in the continuum in photonic waveguides inspired by defects. *Phys. Rev. B* **78**, 075105 (2008).

8. Y. Plotnik, O. Peleg, F. Dreisow, M. Heinrich, S. Nolte, A. Szameit, M. Segev, Experimental observation of optical bound states in the continuum. *Phys. Rev. Lett.* **107**, 183901 (2011).

9. C. W. Hsu, B. Zhen, J. Lee, S.-L. Chua, S. G. Johnson, J. D. Joannopoulos, M. Soljačić, Observation of trapped light within the radiation continuum. *Nature* **499**, 188–191 (2013).

10. J. Gomis-Bresco, D. Artigas, L. Torner, Anisotropy-induced photonic bound states in the continuum. *Nat. Photonics* **11**, 232–236 (2017).

11. S. I. Azzam, V. M. Shalaev, A. Boltasseva, A. V. Kildishev, Formation of bound states in the continuum in hybrid plasmonic-photonic systems. *Phys. Rev. Lett.* **121**, 253901 (2018).

12. S. Mukherjee, J. Gomis-Bresco, P. Pujol-Closa, D. Artigas, L. Torner, Topological properties of bound states in the continuum in geometries with broken anisotropy symmetry. *Phys. Rev. A* **98**, 063826 (2018).

13. H. M. Doeleman, F. Monticone, W. den Hollander, A. Alù, A. F. Koenderink, Experimental observation of a polarization vortex at an optical bound state in the continuum. *Nat. Photonics* **12**, 397–401 (2018).

14. Y. Zhang, A. Chen, W. Liu, C. W. Hsu, B. Wang, F. Guan, X. Liu, L. Shi, L. Lu, J. Zi, Observation of polarization vortices in momentum space. *Phys. Rev. Lett.* **120**, 186103 (2018).

15. A. Cerjan, C. W. Hsu, M. C. Rechtsman, Bound states in the continuum through environmental design. *Phys. Rev. Lett.* **123**, 023902 (2019).

16. J. Jin, X. Yin, L. Ni, M. Soljačić, B. Zhen, C. Peng, Topologically enabled ultrahigh-Q guided resonances robust to out-of-plane scattering, *Nature* **574**, 501–504 (2019).

17. S. Kim, K.-H. Kim, J. F. Cahoon, Optical bound states in the continuum with nanowire geometric superlattices. *Phys. Rev. Lett.* **122**, 187402 (2019).

18. A. C. Overvig, S. C. Malek, M. J. Carter, S. Shrestha, N. Yu, Selection rules for quasibound states in the continuum. *Phys. Rev. B* **102**, 035434 (2020).

19. X. Yin, J. Jin, M. Soljačić, C. Peng, B. Zhen, Observation of topologically enabled unidirectional guided resonances. *Nature* **580**, 467–471 (2020).

20. W. A. Benalcazar, A. Cerjan, Bound states in the continuum of higher-order topological insulators. *Phys. Rev. B* **101**,161116 (2020).

21. A. Cerjan, M. Jürgensen, W. A. Benalcazar, S. Mukherjee, M. C. Rechtsman, Observation of a higher-order topological bound state in the continuum. *Phys. Rev. Lett.* **125**, 213901 (2020).

22. Z. Hayran, F. Monticone, Capturing broadband light in a compact bound state in the continuum. *ACS Photonics* **8**, 813–823 (2021).

23. M. Kang, S. Zhang, M. Xiao, H. Xu, Merging bound states in the continuum at off-high symmetry points. *Phys. Rev. Lett.* **126**, 117402 (2021).

24. S. Vaidya, W. A. Benalcazar, A. Cerjan, M. C. Rechtsman, Point-defect-localized bound states in the continuum in photonic crystals and structured fibers. *Phys. Rev. Lett.* **127**, 023605 (2021).

25. A. A. Yanik, A. E. Cetin, M. Huang, A. Artar, S. H. Mousavi, A. Khanikaev, J. H. Connor, G. Shvets, H. Altug, Seeing protein monolayers with naked eye through plasmonic Fano resonances. *Proc. Natl. Acad. Sci. U.S.A.* **108**, 11784–11789 (2011).

26. B. Zhen, S.-L. Chua, J. Lee, A. W. Rodriguez, X. Liang, S. G. Johnson, J. D. Joannopoulos, M. Soljačić, O. Shapira, Enabling enhanced emission and low-threshold lasing of organic molecules using special Fano resonances of macroscopic photonic crystals. *Proc. Natl. Acad. Sci. U.S.A.* **110**, 13711–13716 (2013).

27. S. Romano, G. Zito, S. Torino, G. Calafiore, E. Penzo, G. Coppola, S. Cabrini, I. Rendina, V. Mocella, Label-free sensing of ultralow-weight molecules with all-dielectric metasurfaces supporting bound states in the continuum. *Photon. Res.* **6**, 726 (2018).

28. K. Hirose, Y. Liang, Y. Kurosaka, A. Watanabe, T. Sugiyama, S. Noda, Watt-class high-power, high-beam-quality photonic-crystal lasers. *Nat. Photonics* **8**, 406–411 (2014).

29. A. Kodigala, T. Lepetit, Q. Gu, B. Bahari, Y. Fainman, B. Kanté, Lasing action from photonic bound states in continuum. *Nature* **541**, 196–199 (2017).

30. B. Wang, W. Liu, M. Zhao, J. Wang, Y. Zhang, A. Chen, F. Guan, X. Liu, L. Shi, J. Zi, Generating optical vortex beams by momentum-space polarization vortices centred at bound states in the continuum. *Nat. Photonics* **14**, 623–628 (2020).

31. C. Huang, C. Zhang, S. Xiao, Y. Wang, Y. Fan, Y. Liu, N. Zhang, G. Qu, H. Ji, J. Han, L. Ge, Y. Kivshar, Q. Song, Ultrafast control of vortex microlasers. *Science* **367**, 1018–1021 (2020).

32. M. Minkov, I. A. Williamson, M. Xiao, S. Fan, Zero-index bound states in the continuum. *Phys. Rev. Lett.* **121**, 263901 (2018).

33. M. Minkov, D. Gerace, S. Fan, Doubly resonant χ(2) nonlinear photonic crystal cavity based on a bound state in the continuum. *Optica* **6**, 1039 (2019).

34. Z. Liu, Y. Xu, Y. Lin, J. Xiang, T. Feng, Q. Cao, J. Li, S. Lan, J. Liu, High-Q quasibound states in the continuum for nonlinear metasurfaces. *Phys. Rev. Lett.* **123**, 253901 (2019).

35. K. Koshelev, S. Kruk, E. Melik-Gaykazyan, J.-H. Choi, A. Bogdanov, H.-G. Park, Y. Kivshar, Subwavelength dielectric resonators for nonlinear nanophotonics. *Science* **367**, 288–292 (2020).

36. J. Wang, M. Clementi, M. Minkov, A. Barone, J.-F. Carlin, N. Grandjean, D. Gerace, S. Fan, M. Galli, R. Houdré, Doubly resonant second-harmonic generation of a vortex beam from a bound state in the continuum. *Optica* **7**, 1126 (2020).

37. J. S. Ginsberg, A. C. Overvig, M. M. Jadidi, S. C. Malek, G. N. Patwardhan, N. Swenson, N. Yu, A. L. Gaeta, Enhanced harmonic generation in gases using an all-dielectric metasurface. *Nano* **10**, 733–740 (2020).

38. S. Campione, S. Liu, L. I. Basilio, L. K. Warne, W. L. Langston, T. S. Luk, J. R. Wendt, J. L. Reno, G. A. Keeler, I. Brener, M. B. Sinclair, Broken symmetry dielectric resonators for high quality factor fano metasurfaces. *ACS Photonics* **3**, 2362–2367 (2016).

39. K. Koshelev, S. Lepeshov, M. Liu, A. Bogdanov, Y. Kivshar, Asymmetric metasurfaces with high-Q resonances governed by bound states in the continuum. *Phys. Rev. Lett.* **121**, 193903 (2018).

40. K. Koshelev, A. Bogdanov, Y. Kivshar, Meta-optics and bound states in the continuum. *Sci. Bull.*, **64**, 836–842 (2019).

41. G. Zito, S. Romano, S. Cabrini, G. Calafiore, A. C. D. Luca, E. Penzo, V. Mocella, Observation of spin-polarized directive coupling of light at bound states in the continuum. *Optica* **6**, 1305–1312 (2019).

42. M. V. Gorkunov, A. A. Antonov, Y. S. Kivshar, Metasurfaces with maximum chirality empowered by bound states in the continuum. *Phys. Rev. Lett.* **125**, 093903 (2020).

43. Y. Kurosaka, S. Iwahashi, Y. Liang, K. Sakai, E. Miyai, W. Kunishi, D. Ohnishi, S. Noda, On-chip beam-steering photonic-crystal lasers. *Nat. Photonics* **4**, 447–450 (2010).

44. G. von Freymann, A. Ledermann, M. Thiel, I. Staude, S. Essig, K. Busch, M. Wegener, Three-dimensional nanostructures for photonics. *Adv. Funct. Mater.* **20**, 1038–1052 (2010).

45. J. K. Hohmann, M. Renner, E. H. Waller, G. von Freymann, Three-dimensional μ-printing: An enabling technology. *Adv. Opt. Mater.* **3**, 1488–1507 (2015).

46. J. Li, B. Jia, G. Zhou, M. Gu, Fabrication of three-dimensional woodpile photonic crystals in a PbSe quantum dot composite material. *Opt. Express* **14**, 10740–10745 (2006).

47. J. D. Joannopoulos, S. G. Johnson, J. N. Winn, R. D. Meade, *Photonic Crystals: Molding the Flow of Light (Second Edition)* (Princeton Univ. Press, 2011).

48. D. B. Fullager, G. D. Boreman, T. Hofmann, Infrared dielectric response of nanoscribe IP-dip and IP-L monomers after polymerization from 250 cm^{-1} to 6000 cm^{-1}. *Opt. Mater. Express* **7**, 888 (2017).

49. A. F. Oskooi, D. Roundy, M. Ibanescu, P. Bermel, J. D. Joannopoulos, S. G. Johnson, Meep: A flexible free-software package for electromagnetic simulations by the FDTD method. *Comput. Phys. Commun.* **181**, 687–702 (2010).

50. S. G. Johnson, J. D. Joannopoulos, Block-iterative frequency-domain methods for Maxwell's equations in a planewave basis. *Opt. Express* **8**, 173–190 (2001).

51. V. Liu, S. Fan, S4: A free electromagnetic solver for layered periodic structures. *Comput. Phys. Commun.* **183**, 2233–2244 (2012).

52. T. Inui, Y. Tanabe, Y. Onodera, *Group Theory and Its Applications in Physics* (Springer Series in Solid-State Sciences, Springer-Verlag, 1990).

53. A. Taghizadeh, I.-S. Chung, Quasi bound states in the continuum with few unit cells of photonic crystal slab. *Appl. Phys. Lett.* **111**, 031114 (2017).
